# Resolving the complete genome of *Kuenenia stuttgartiensis* from a membrane bioreactor enrichment using Single-Molecule Real-Time sequencing

**DOI:** 10.1038/s41598-018-23053-7

**Published:** 2018-03-15

**Authors:** Jeroen Frank, Sebastian Lücker, Rolf H. A. M. Vossen, Mike S. M. Jetten, Richard J. Hall, Huub J. M. Op den Camp, Seyed Yahya Anvar

**Affiliations:** 1 Soehngen Institute of Anaerobic Microbiology, Radboud University Nijmegen, Nijmegen, The Netherlands; 2 Department of Microbiology, IWWR, Radboud University Nijmegen, Nijmegen, The Netherlands; 3 Leiden Genome Technology Center, Leiden University Medical Center, Leiden, The Netherlands; 4 Pacific Biosciences, Menlo Park, California United States of America; 5 Department of Human Genetics, Leiden University Medical Center, Leiden, The Netherlands

## Abstract

Anaerobic ammonium-oxidizing (anammox) bacteria are a group of strictly anaerobic chemolithoautotrophic microorganisms. They are capable of oxidizing ammonium to nitrogen gas using nitrite as a terminal electron acceptor, thereby facilitating the release of fixed nitrogen into the atmosphere. The anammox process is thought to exert a profound impact on the global nitrogen cycle and has been harnessed as an environment-friendly method for nitrogen removal from wastewater. In this study, we present the first closed genome sequence of an anammox bacterium, *Kuenenia stuttgartiensis* MBR1. It was obtained through Single-Molecule Real-Time (SMRT) sequencing of an enrichment culture constituting a mixture of at least two highly similar *Kuenenia* strains. The genome of the novel MBR1 strain is different from the previously reported *Kuenenia* KUST reference genome as it contains numerous structural variations and unique genomic regions. We find new proteins, such as a type 3b (sulf)hydrogenase and an additional copy of the hydrazine synthase gene cluster. Moreover, multiple copies of ammonium transporters and proteins regulating nitrogen uptake were identified, suggesting functional differences in metabolism. This assembly, including the genome-wide methylation profile, provides a new foundation for comparative and functional studies aiming to elucidate the biochemical and metabolic processes of these organisms.

## Introduction

Anaerobic ammonium-oxidizing (anammox) bacteria are a group of slow growing, strictly anaerobic chemolithoautotrophic microorganisms affiliated with the order *Brocadiales* of the phylum *Planctomycetes*^1,2^. These microorganisms are characterized by their capability to oxidize ammonium to nitrogen gas using nitrite as a terminal electron acceptor^3,4^. Recent studies have demonstrated that catalytic reactions of the anammox pathway, including hydrazine synthesis and oxidation^5^, occur in a unique intracellular membrane-bound organelle termed the anammoxosome. This organelle comprises 50–70% of the cell volume and is surrounded by a membrane containing distinctive ladderane lipids^6–9^.

Anammox bacteria have been detected in nearly all anoxic environments that contain fixed nitrogen^10^. These include natural ecosystems like soil^11^, freshwater and marine sediments, oxygen minimum zones^12–14^, and engineered environments such as wastewater treatment plants^15^. In these habitats, anammox bacteria facilitate the release of fixed nitrogen into the atmosphere. It is estimated that up to 50% of all released nitrogen gas is produced by these microorganisms, thereby exerting a significant impact on the global nitrogen cycle^16,17^. In addition to its function as a nitrogen sink in natural environments, the anammox process has been successfully implemented as a sustainable and efficient method for removal of nitrogen from wastewater^18^.

Fifteen anammox species representing five different genera have been described since the first identification of an anammox bacterium in 1999^15^. 16S rRNA gene-based analyses of environmental samples indicate a considerably greater and mostly uncharacterized anammox biodiversity^10^. Thus far, all species have resisted cultivation using conventional techniques. Enrichment cultures for nine anammox species have been obtained through continuous culturing in bioreactor systems under substrate limitation^10^. Many metagenome sequencing efforts have led to genome assemblies for eight different anammox species^1,19–25^, none of which have been fully closed, primarily due to limitations of the sequencing technologies used.

The first assembly of an anammox genome was that of the freshwater species *Kuenenia stuttgartiensis* in 2006^1^. In this study, an enrichment culture obtained by inoculating a gas lift bioreactor with sludge from the nitrification stage of a wastewater treatment plant was used. Genomic DNA was extracted followed by Sanger sequencing of whole-genome shotgun DNA libraries. Numerous gaps in the assembly were closed with the aid of BAC and fosmid clones. Ultimately five scaffolds were constructed with a total size of approximately 4.2 Mb. After five years of continuous cultivation, the same enrichment culture was re-sequenced in 2009 using Illumina GAIIx, which further improved the original assembly and closed one of the remaining gaps^20^.

It is remarkably challenging to completely assemble and close a genome relying exclusively on data produced by 2^nd^ generation sequencing platforms. Biases and artifacts introduced during inherent DNA amplification steps often lead to fragmented genome coverage. In addition, the relatively short read lengths prevent the resolution of large genomic repeats, highly similar paralogs and other structural variations by the assembler, leaving the assembly incomplete. Advanced 3^rd^ generation sequencing technologies, such as Pacific Biosciences (PacBio) single-molecule real-time (SMRT) sequencing and Oxford Nanopore DNA sequencing, do not require DNA amplification and generate long, multi-Kb reads. This enables the resolution of large structural variations, significantly reducing the level of complexity in *de novo* assembly approaches. Furthermore, SMRT sequencing is essentially free of context-specific biases and allows inference of DNA methylation through observation of polymerase kinetics during sequencing^26,27^.

In this study, we present the closed genome sequence and the genome-wide methylation profile of *Kuenenia stuttgartiensis* MBR1, a novel *Kuenenia* strain growing as suspended planktonic cells in a membrane bioreactor. This entirely new assembly represents the first completely closed anammox genome. Together with the methylome, it will provide a new foundation for prospective comparative and functional analysis aiming to elucidate the intricate biochemistry, metabolism and genomic versatility of these unique microorganisms.

## Results and Discussion

### Genome assembly and annotation

DNA was extracted from a highly enriched (~95% of total bacterial biomass^28^) planktonic culture of *Kuenenia stuttgartiensis* sustained in an anoxic membrane bioreactor and sequenced using the PacBio RSII SMRT sequencing platform. Over the course of multiple sequencing runs, 540,044 single-molecule long reads were obtained (14 SMRT cells, Supplementary Table S1). Inherent to the SMRT sequencing technology, unprocessed reads manifested a relatively low accuracy due to the presence of randomly distributed sequencing errors^26^. These errors were corrected using the hierarchical genome-assembly process (HGAP) pipeline^29^, yielding 108,054 highly accurate consensus sequences that ranged from 500 bp to over 27 Kb in length with a median length of 2,558 bp (Supplementary Table S1).

An initial *de novo* assembly of the metagenome, using the entire dataset of corrected reads, resulted in 135 contigs spanning 8.4 Mb. The assembly graph was inspected to assess the structure of the assembly (Supplementary Figure S1). This revealed deeply covered, fully assembled regions interrupted by numerous unresolved regions, depicted in the graph as bubbles. These assembly bubbles indicated the presence of structural variations and consistently showed very distinctive coverage patterns, containing both high and low coverage paths. This observation suggested the presence of at least two highly similar strains at different levels of abundance.

**Figure 1:**
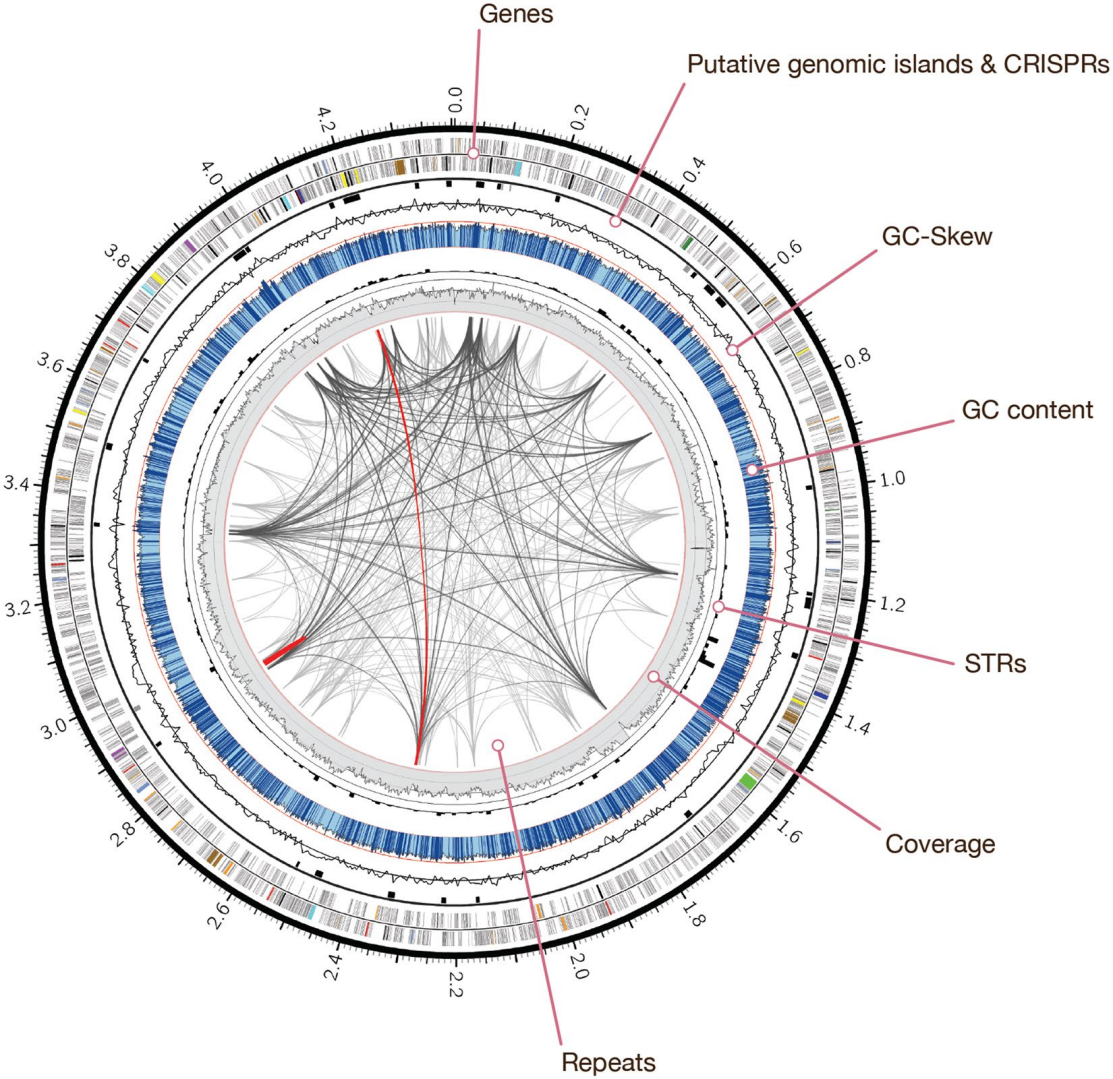
Genome composition of *Kuenenia stuttgartiensis* MBR1. Circos plot illustrating the major features of the *K. stuttgartiensis* MBR1 genome. The outermost ring shows genes on the forward (2,071) and reverse (2,022) strand, highlighting specific genes of interest (see legend below). Putative genomic islands (black) and CRISPRs (grey) are outlined next, followed by a line plot that draws the GC skew. The GC-content is shown in blue, with light blue regions indicating <5% and dark blue >5% deviation from the mean (bin size: 1 Kb). The red line indicates GC% 50. Short tandem repeats (260) are indicated by a black histogram (bin size: 10 Kb). The innermost grey ring illustrates the SMRT sequencing depth (bin size: 1 Kb, min: 118.6×, max shown: 600×). The links inside represent repetitive sequences and structural variations within the genome that demonstrate >99% similarity at the nucleotide level. Repeats >2 Kb (294) are colored light grey, >3 Kb (137) dark grey and repeats >5 Kb (2) are colored red. Highlighted genes: hydroxylamine oxidoreductases (red), hydrazine synthases (purple), nitric oxide and nitrite reduction (dark green), *bc*_1_ complexes (dark blue), ATP synthesis (turquoise), substrate uptake and substrate trafficking (light blue), CO_2_ fixation (brown), nitrite oxidation (bright green), S-layer proteins (yellow), transposases (black), restriction-modification systems (orange).

To obtain separate assemblies of both strains, uncorrected reads were assigned to metagenome bins based on coverage depth (Supplementary Figure S2). Reads aligning to regions with high coverage (>110×) were used to assemble the dominant strain. For the less abundant strain, reads aligning to low coverage regions (25–120×) and regions with extremely high coverage (>225×) were selected. The inclusion of highly abundant regions in both bins ensured that shared, conserved genomic regions are used for the assembly of both strains. The high and low coverage bins were error corrected and assembled separately, yielding 66 (5.7 Mb) and 157 (7.7 Mb) contigs, respectively (Supplementary Figure S3). Subsequently, we focused on assembling the dominant strain contained in the high coverage bin. Further refinement of the coverage-based binning approach yielded 48 contigs, including seven contigs that had a markedly higher (>190×) coverage depth (Supplementary Figure S4). A scaffolding and gap-filling procedure of the highly covered contigs resulted in the complete genome contained in one contig. The genome was circularized, resulting in a single chromosome sequence of 4,406,153 bp in length, with an average GC-content of 41.1% (Fig. 1, Table 1). We refer to this closed genome as the “MBR1” genome, reflecting its origin from planktonic cells sustained in a membrane bioreactor.

**Table 1:** Genome assembly overview.

	MBR1 strain (2014)	Low abundance assembly (2014)	KUST strain (2006)
Sequencing technology	SMRT sequencing	SMRT sequencing	Sanger
Number of scaffolds	1	157	5
Bases in scaffolds	4,406,153 bp	7,726,594 bp	4,218,325 bp
Mean sequencing depth	419×	240×	22×
GC-content	41.1%	41.0%	41.0%

Next, we performed an independent sequencing run to assess the accuracy and validity of the assembly (Supplementary Table S1, Supplementary Figure S5). The concordance between corrected reads and the genome sequence was over 99.99%, with no indication of sequence disagreement. In total, 322 long reads spanned the location were the genome was circularized without gaps, indicating the correctness and reliability of the closed genome sequence.

The MBR1 genome contained many repetitive sequences, including two large repeat structures, each over 5 Kb in length. In addition, 260 short tandem repeats were found (Fig. 1). The GC-content was mostly uniform, but nonetheless revealed 823 regions (1 Kb window) that deviated at least 5% from the mean. Algorithms analyzing sequence composition such as dinucleotide bias, codon usage and the presence of indicator genes predicted 27 putative genomic islands (GIs), most of which had a distinct GC-content (Supplementary Table S2, Fig. 1). Automated annotation was performed using Prokka^30^ and the MicroScope integrated annotation platform^31^, followed by manual curation. Although most genes located in GIs encoded hypothetical proteins, they also contained transposases and secretion pathway proteins (ExeA). In total 4,093 genes were predicted, with an average size of 876 bp. These include one complete operon encoding the ribosomal RNAs (16S, 23S, 5S), one 6S rRNA gene, one tmRNA, the small and large signal recognition particle RNAs and 45 tRNA genes (Table 2). Coding density reached 81.2% with 46.7% of the genes having an automatic function prediction. Seven clustered regularly interspaced short palindromic repeat (CRISPR) regions were detected, four of which were positioned closely together in a region 8.8 Kb in size (position 489,976–498,766), located approximately 12 Kb upstream of a CRISPR-associated Cas6 gene (Supplementary Table S3).

**Table 2:** Annotation overview.

	MBR1 strain (2014)	KUST strain (2006)*
Number of CDS	4,044	3,772
Average gene length	876 bp	902 bp
Coding density	81.2%	81.7%
Genes with functional assignments	1,912 (46.7%)	1,446 (38.3%)
Number of rRNAs	3 (16S-23S-5S)	3 (16S-23S-5S)
Number of tRNAs	45 + 1 tmRNA	45 + 1 tmRNA

*The KUST genome was re-annotated using Prokka to ensure fair comparisons could be made with the MBR1 strain.

### Genome methylation state

SMRT sequencing enables the inference of adenine and cytosine methylation for every incorporated base through real-time observation of DNA polymerase kinetics. This allowed us to report for the first time the complete methylome of an anammox bacterium. The annotated genome of the MBR1 strain revealed a diversity of restriction modification systems and putative DNA methylases (Supplementary Tables S4 + S5), suggesting SMRT sequencing should be able to detect various types of methylation. Since the genomic DNA did not receive Tet1 oxidation treatment prior to sequencing, only N6-methyladenine (6mA) and 4-methylcytosine (4mC) signals could be reliably detected^32^. The threshold for reliable identification of methylated bases was set guided by the distribution of modification quality values (Supplementary Figure S8). Adenine and cytosine bases displayed a distinct modification signal that corresponded robustly to the overall coverage depth on each strand (Supplementary Figure S8). The genome was found to be highly methylated, containing 28,211 6mA and 9,128 4mC base modifications that were distributed throughout the genome (Fig. 3). These modifications may protect the genome from damage during degradation of foreign DNAs using restriction enzymes. Using sequence context analysis, 27,012 methylated adenines (95.7%) and 7,963 methylated cytosines (87.2%) were associated with six and three putative sequence recognition motifs, respectively (Table 3). All nine motifs reside predominantly in coding regions of the genome and occur mainly in their methylated state (83.0%). Certain methylated sites might be involved in regulation of gene expression, but we failed to observe distinct distribution patterns indicating this. The six sequence motifs associated with adenine methylation were methylated in>95% of all occurrences. The three cytosine methylation motifs showed significantly lower rates of methylation, on average 55.0%. These patterns likely reflect the quality of the signal and precision in detection rather than true variation in methylation.

**Table 3:** Adenine and cytosine methylation sequence motifs in the MBR1 strain.

Motif	# Motifs detected	# Motifs methylated	% Motifs methylated	% Intergenic	Mean Coverage
G^**m6**^**A**TC	16,942	16,909	99.8	10.5	198.3
G_2_HN^**m4**^**C**C	9,489	4,397	46.3	9.7	207.2
G_2_NC^**m4**^**C**W	4,253	2,822	66.3	7.2	188.9
C_2_^**m6**^**A**YC_2_	3,453	3,437	99.5	14.5	196.8
GCRC^**m6**^**A**G	2,937	2,827	96.2	12.4	199.0
CTRG^**m6**^**A**G	1,637	1,621	99.0	14.1	192.8
GCT^**m6**^**A**TC	1,520	1,515	99.7	12.5	199.5
GACC^**m4**^**C**T	1,160	744	64.1	16.4	178.1
CHC_2_^**m6**^**A**C_2_D	738	703	95.3	7.7	199.6

Motifs with a modification quality value ≥100 were considered. Methylated bases are in bold.

### Comparative genome analysis

**Figure 2:**
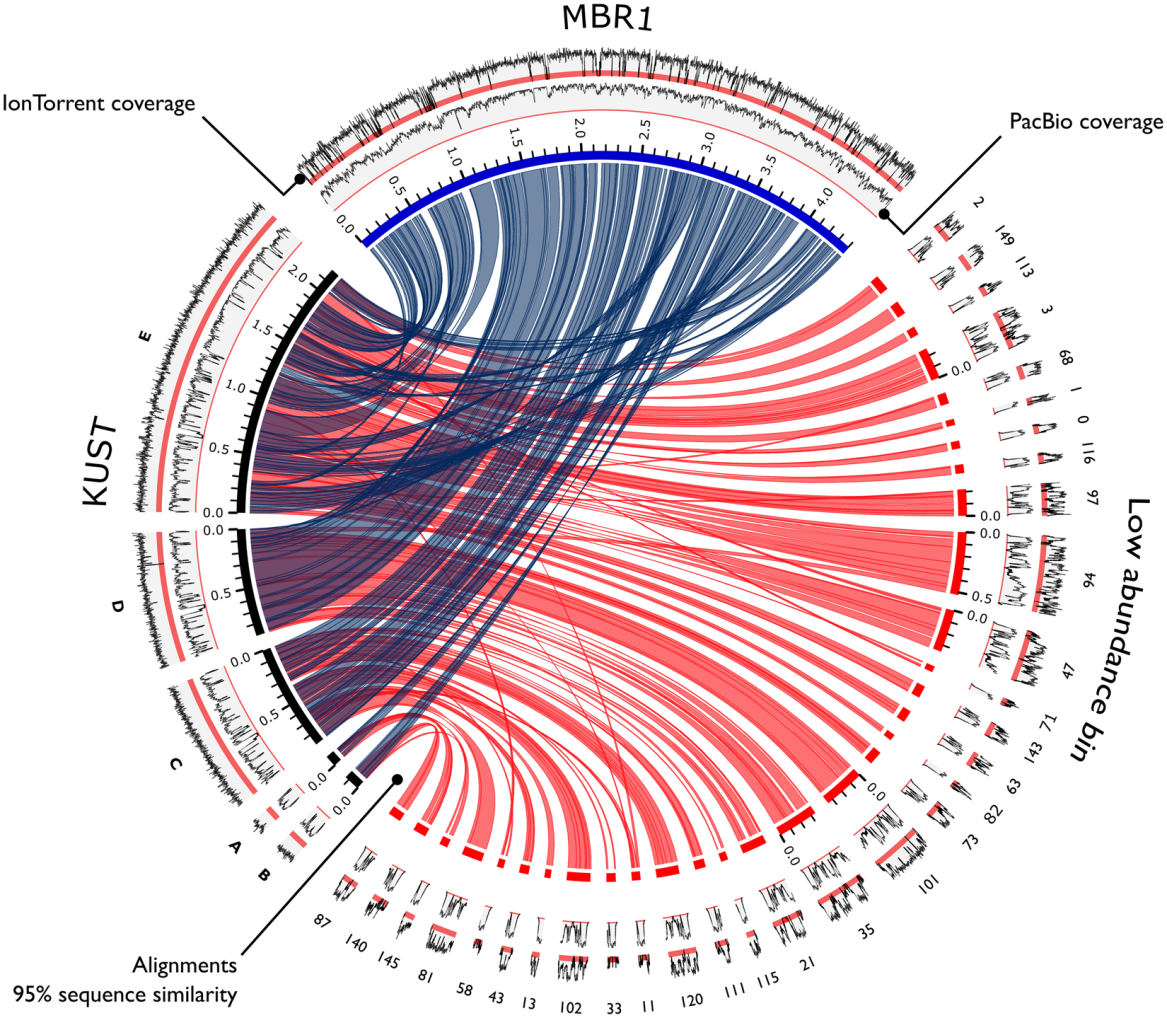
Whole-genome alignments of *Kuenenia* assemblies. Circos plot demonstrating the sequence similarity for the closed MBR1 (blue) genome, 2006 KUST (black) genome, and the KUST genome versus the assembly of the low abundance bin (red). Links represent >95% sequence similarity at the nucleotide level. Contigs <50 Kb and alignments <5 Kb are excluded to enhance figure clarity. Note that KUST scaffolds “C” and “D” have been reversed. The two outermost tracks display the sequencing depth of the PacBio SMRT sequencing (2014, inner track) and IonTorrent resequencing (2012, outer track). Red bands emphasize low coverage regions (<25×). Coverage tracks are normalized and do not scale to the absolute sequencing depth.

We compared the assembly of the closed MBR1 genome to the 2006 *K. stuttgartiensis* reference genome (hereafter referred to as “KUST”). The MBR1 genome was over 187 Kb larger in size. Sequence variation between the assemblies was small, with an average nucleotide identity (OrthoANI) of 99.40% (Supplementary Figure S6). The 16S rRNA genes of both genomes were identical and gene order was mostly conserved. Despite high sequence similarity, whole-genome alignments exposed many large genomic rearrangements, including 168 relocations, 31 translocations, 29 inversions and other structural variations (Fig. 2). Furthermore, only 86.48% of the MBR1 genome aligned to the KUST genome at ≥95% nucleotide identity (Supplementary Figure S6), suggesting specific regions have been lost, gained, or otherwise changed in one genome versus the other. Together with the distinctive coverage patterns observed during metagenome binning, these findings suggest that the MBR1 genome represents a novel *Kuenenia* strain that is distinct from the 2006 KUST strain.

**Figure 3:**
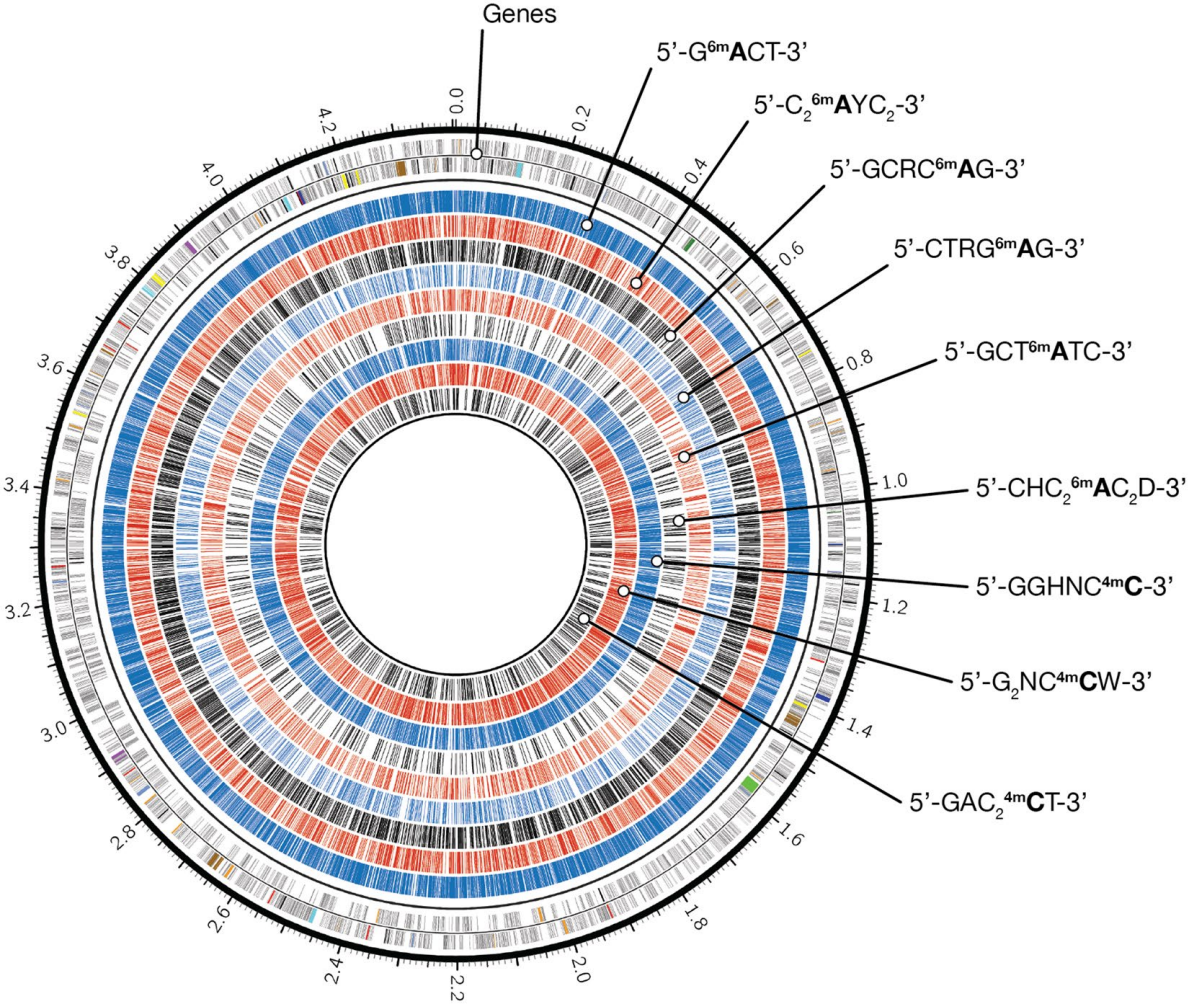
Genome-wide methylation profile of *Kuenenia stuttgartiensis* MBR1. Circos plot showing the global methylation state of the *K. stuttgartiensis* MBR1 genome. The outermost ring shows the genes on the forward and reverse strand. The subsequent tracks represent the distribution of methylated adenine and cytosine bases that are associated with methyltransferase recognition motifs. From outside to inside: 5′-G^m6^ATC-3′ (blue), 5′-C_2_^m6^AYC_2_–3′ (red), 5′GCRC^m6^AG-3′ (black), 5′-CTRG^m6^AG-3′ (blue), 5′-GCT^m6^ATC-3′ (red), 5′-CHC_2_^m6^AC_2_D-3′ (black), 5′-GGHNC^m4^C-3′ (blue), 5′-G_2_NC^m4^CW-3′ (red) and 5′-GAC_2_^m4^CT-3′ (black).

Analysis of the 157 contigs (7.7 Mb) in the low coverage bin revealed that the MBR1 genome was mostly contained, with 97.0% of its genome aligning to the contigs at ≥95% nucleotide identity. Scaffolding of these contigs against the MBR1 genome produced two scaffolds constructed out of 70 contigs, spanning 94% of the MBR1 genome. Contrastingly, the KUST genome was found almost completely present in the low coverage bin, with 99.3% of its genome aligning to the low abundant contigs (Fig. 2, Supplementary Figure S6). Scaffolding yielded five scaffolds covering 98% of the KUST genome. The scaffolds consisted of 71 contigs, including 26 contigs that were not used for scaffolding the MBR1 genome. These contigs largely made up the genome of the low abundant strain we separated from the MBR1 strain through coverage binning. The bin contained an additional megabase of sequence data organized in 60 contigs that could not be scaffolded. Taxonomic classification of these contigs demonstrated high sequence similarity to *Kuenenia*, possibly indicating even greater strain variability present in the dataset. We were not able to fully separate and close the KUST genome present in the low coverage bin. Additional sequencing would be required to completely assemble the genomes of the other strains present in the culture.

All uncorrected reads (647,491 reads, 15 SMRT cells) were mapped to the MBR1 genome, the KUST genome and the low coverage bin to further examine the differences observed in the whole-genome alignments. In total, 536,929 (82.9%) of the reads aligned to the dominant MBR1 genome, establishing a mean sequencing depth of 419×. The coverage never dropped below 100× and was in full agreement with the structure of the assembled genome. The KUST genome was not as well supported by the SMRT sequencing data and demonstrated wildly fluctuating coverage levels containing many large drops in sequencing depth. In total, 521,716 (80.6%) of the reads mapped to the KUST genome, generating an average coverage of 394.9×. We identified 115 regions ranging from 50 to 161 bp in size in the KUST genome that had a coverage below <25×. The assembly of the low coverage bin showed highly alternating coverage levels that reflected its heterogeneous nature, namely the presence of deeply covered regions shared between strains, regions that are unique to one of the two identified strains, and other strain variations that still remained uncharacterized.

To further characterize strain diversity, we compared the protein coding sequences of the MBR1 and KUST strains using BLASTP. The KUST genome was re-annotated using Prokka to ensure comparable gene predictions. In total, 3,095 best bi-directional hits were identified at 90% identity level, corresponding to 76.53% and 82.05% of all proteins in the MBR1 (4,044 CDS) and KUST (3,772 CDS) genomes, respectively. Eleven genes encoding restriction-modification and DNA and RNA methylation systems present in strain MBR1 were conserved in the KUST genome (Supplementary Tables + S5), implying that the KUST genome may exhibit similar methylation patterns as detected in this study. BLAST results were further processed to filter out small and low identity (<90%) alignments by excluding alignments with ≤75% or ≥120% coverage of the query sequence. We identified 562 proteins (13.9%) distributed throughout the MBR1 genome that did not have a significant hit to the KUST genome. In turn, 428 (11.4%) proteins on the KUST genome had no hit to MBR1 (Supplementary Figure S7, Supplementary File S1). Among the most interesting findings is the discovery of a putative type 3b (sulf)hydrogenase in MBR1 (KSMBR1_3671-KSMBR1_3674). This multifunctional enzyme complex is involved in hydrogen cycling. The beta and gamma subunits are able to catalyze the oxidation of hydrogen, but also the reduction of elemental sulfur to hydrogen sulfide^33^. This suggests strain MBR1 might be able to use sulfur compounds as electron acceptors, which seems unlikely in the context of the anammox metabolism. Furthermore, a complete duplication of the hydrazine synthesis gene cluster was identified (*hzsABC*, KSMBR1_3603-KSMBR1_3601 and KSMBR1_2713-KSMBR1_2711), in addition to a partial copy containing only subunits *hzsB* and *C* (KSMBR1_2704, KSMBR1_2703). Hydrazine synthase is a slow enzyme, which might explain the long doubling time of anammox bacteria^34^. Multiple copies of this gene cluster may facilitate increased levels of transcription and translation and thus elevated turnover of ammonium due to a gene-dose effect. This could increase the rate of anammox metabolism, possibly leading to a faster growth rate. Furthermore, four putative ammonium transporters and proteins regulating nitrogen uptake were identified (Supplementary File S1), suggesting functional differences in ammonium transport and metabolism between the two *Kuenenia* strains. In addition, 72 putative proteins involved in DNA modification and 34 proteins presumably taking part in outer membrane biosynthesis were found to be unique to strain MBR1, indicating membrane modifications by this *Kuenenia* strain, possibly to evade phage invasion. Finally, many genes involved in flagellar biosynthesis show signs of degradation in comparison to KUST, implying strain MBR1 may have lost functional flagella. Genes exclusive to the KUST genome are mainly involved in outer membrane biosynthesis and DNA modification systems (Supplementary File S1). However, additional studies on anammox physiology and biochemistry are required to examine the impact of these genomic variations.

### Detection of multiple *Kuenenia* strains

The detection of multiple *Kuenenia* strains was unexpected, notably since previous studies have not reported strains other than the one represented by the KUST genome. Strous *et al*. obtained the enrichment of *K. stuttgartiensis* by inoculating a gas lift bioreactor with nitrifying activated sludge and sequenced this culture in 2002^1^. The absence of single nucleotide polymorphisms in the assembly suggested that only a single *K. stuttgartiensis* strain was present^1^. In 2004, the culture was transferred into a sequencing fed-batch reactor. Speth *et al*. sequenced this culture using Illumina GAIIx in 2009, following five years of continuous cultivation. Results of this study confirmed the integrity and stability of the original KUST genome, with no indication of strain variations^20^. The current culturing setup was established after the 2009 re-sequencing and utilizes a membrane bioreactor that was inoculated with enriched anammox biomass from the original sequencing fed-batch reactor^35^. The most recent re-sequencing effort prior to SMRT sequencing (2014) was performed in 2012, utilizing the IonTorrent sequencing platform (BioProject accession: PRJEB4259, SRA accession: ERR342261).

We re-analyzed the IonTorrent dataset to search for evidence of multiple strains. In total 93% of the reads mapped to the MBR1 strain, generating a very variable coverage profile that contained many drops in coverage (Fig. 2). We identified 235.7 Kb of sequence without coverage, equivalent to 5.3% of the genome. The KUST genome was well supported by the data, with 96.3% of all reads aligning to the genome. Virtually every base of the genome was covered and the coverage profile was uniform (Fig. 2). Subsequently, we investigated genes exclusive to the MBR1 (562) and KUST (428) genomes. Both gene sets were blasted (BLASTn) against the IonTorrent and SMRT sequencing datasets (Supplementary Figure S9). This revealed the absence of 140 genes unique to MBR1 in the IonTorrent data. In addition, 204 genes were only partially found, with >25% of the gene sequence uncovered. The KUST gene set was well supported by the IonTorrent data. We could not detect *Kuenenia* strains other than the one represented by the KUST genome in this dataset. This is in contrast to the SMRT sequencing data, which covered the complete MBR1 gene set and asserted its dominance over the KUST genome (Supplementary Figure S9).

Within a period of two years (between the IonTorrent and PacBio sequencing in 2012 and 2014, respectively), we observed the emergence of a new *Kuenenia* strain that quickly established dominance, outcompeting the KUST strain that had been stable for many years^20^. This strongly suggests multiple *Kuenenia* strains have been present in the culture since its inoculation. We hypothesize the change in cultivation conditions has caused the MBR1 strain to become dominant. This idea is supported by the near complete presence of the KUST genome in the low coverage bin. We suspect this shift was induced during the transition from a sequencing batch reactor to a membrane bioreactor in 2009. This change in cultivation enabled obtaining planktonic cells instead of flocs which resulted in an enrichment of up to 95% of the total bacterial biomass^28^. Possibly, this induced the selective pressure driving the change in culture composition. Although the 2012 IonTorrrent re-sequencing data did not provide evidence for multiple strains, the MBR1 strain could already have been positively selected for, but still was below the detection limit due to its long generation time^34^. This is plausible since anammox bacteria proliferate slowly, doubling on average once a week. Ultimately, this gradual change would lead to the shift in composition that was uncovered with SMRT sequencing in 2014. Other factors exerting selective pressure seem unlikely to have caused the change, due to aforementioned proliferation time of *Kuenenia* and the stable conditions in the bioreactor. External contamination in the laboratory could explain the sudden appearance of a novel strain and cannot be fully ruled out.

Several factors may have hindered the identification of *Kuenenia* species other than the KUST strain. It is clear that the KUST strain was by far most abundant during earlier sequencing studies, challenging the detection of other strains. In addition, traditional sequencing methods provide relatively small read lengths and generate biased datasets as a result of DNA amplification steps, constraining the assembly of highly similar strains. In contrast, SMRT sequencing does not require DNA amplification and generates long single-molecule reads that are virtually free of context-specific biases. This enabled us to distinguish and assemble two highly similar *Kuenenia* strains and to successfully close the genome of the novel and dominant MBR1 strain.

## Methods

### Genomic DNA preparation and sequencing

DNA was extracted for SMRT sequencing in March 2014 from planktonic cells obtained out of a highly enriched culture (~95% of total bacterial biomass^28^) of *Kuenenia stuttgartiensis*. The single cells have been sustained in an anoxic membrane bioreactor since 2009 and were originally acquired from flocs of cells that have been in continuous culture since 2002^1^. Setup and operation of the anoxic membrane bioreactor has been described in detail by Kartal *et al*.^35^. Genomic DNA was isolated and fragmented with G-tubes (Covaris). SMRTbell DNA template libraries (insert size of ~20 Kb) were prepared according to the manufacturer’s specification followed by size selection using Sage science’s BluePippin to remove short molecules. SMRT sequencing (15 SMRT cells) was performed on the Pacific Biosciences RS II sequencer according to standard protocols using MagBead loading with P4-C2/P5-C3 sequencing chemistry and 1 × 180 minutes movie-time. Sequencing of several SMRT cells was suboptimal, generating few usable reads.

### *De novo* genome assembly

Starting with 14 SMRT cells, 540,044 uncorrected continuous reads longer than 500 bp with a quality value over 0.75 were obtained and merged together into a single dataset (Supplementary Table S1). Random errors in long seed reads were corrected using the hierarchical genome-assembly process (HGAP) pipeline^29^ (seed length cutoff: 2 Kb, SMRTanalysis v.2.3.0). This produced 108,054 long, corrected reads. An initial *de novo* assembly of the metagenome was performed using Celera Assembler 8.1^36^ using all corrected reads, yielding 135 contigs in total (assembler configuration settings are provided in Supplementary Data S1). All uncorrected reads were mapped back to the assembled metagenome using BLASR^37^ (available in SMRTanalysis v.2.3.0), resulting in the alignment of 419,749 reads (77.7%). Aligned reads were subsequently assigned to different metagenome bins based on the coverage depth of the genomic region they aligned too. Coverage cutoffs were set guided by examination of assembly graphs rendered with Gephi 0.8.2^38^ (Supplementary Figure S1) and histograms of the coverage depth (Supplementary Figure S2). Uncorrected reads aligning to highly covered regions (>110×, 179,021 reads (33.1%) referred to as “high coverage bin”) and reads aligning to low covered or very highly covered regions (25–120× and >225×, 155,794 reads (28.8%); referred to as “low coverage bin”) were separately corrected using HGAP (seed length cutoff: 2 Kb) and assembled. The inclusion of very deeply covered regions in both bins ensured shared genomic regions were assembled for both strains. In total 66 and 157 contigs were assembled for the high and low coverage bin respectively (Supplementary Table S2).

The high coverage bin was refined by excluding 3,085 reads that aligned to regions on the assembly of the high coverage bin with less than 100-fold coverage (Supplementary Figure S3). The remaining reads (175,936 reads, 32.6%) were corrected (HGAP seed length cutoff: 4 Kb) and assembled resulting in 48 contigs. Seven contigs (total size ~4.46 Mb) showed markedly higher coverage depth (>190-fold, Supplementary Figure S3). PBJelly (PBSuite 14.9.9)^39^ was used to perform scaffolding and subsequent gap-filling on this set of contigs employing all reads in the high coverage bin, producing five contigs total. Manual inspection of the five contigs using Mauve^40^ revealed repeated sequences at the ends of each scaffold. Repeated regions plus an additional kilobase of sequence were clipped after which another round of scaffolding and gap-filling was initiated. Scaffolding of the five trimmed contigs yielded the complete genomic sequence of the MBR1 strain in one continuous piece. The ends of the assembled sequence were manually inspected and clipped to circularize the genome. One additional SMRT cell was sequenced to aid in the assessment of the assembly in terms of the accuracy and validity, and to facilitate the detection of base modifications at higher levels of confidence.

### Annotation

Automatic annotation of the MBR1 genome was performed using Prokka 1.10^30^. A collection of annotated proteins from the 2006 KUST genome^1^ was supplied to Prokka as a trusted source for annotation. The generated annotation was augmented with annotation produced by the MicroScope integrated annotation platform^41^. In addition, a set of genes of particular interest was verified and curated manually. The 2006 KUST genome was re-annotated using Prokka to enable unbiased comparisons with the closed MBR1 genome. Genomic repeats and other structural variations were identified using NUCmer 3.1 (part of MUMmer 3.23^42^). Tandem repeats were identified using the Tandem Repeat Finder online service^43^. The genome was analyzed for the presence of CRIPSRs using the CIRSPRFinder web tool^44^. Genomic islands were inferred using IslandViewer 3^45^, which integrates results of three prediction methods: SIGI-HMM^46^, IslandPath-DIMOB^47^ and IslandPick^48^.

### Comparative genomics

Average nucleotide identities (ANI) were calculated using the OrthoANI method^49^. Whole-genome alignments were generated using NUCmer 3.1 (MUMmer 3.23^42^). Best bi-directional hits (BBHs) were identified using BLASTP 2.4.0+^50^ and a custom python script. Contigs in the low coverage bin were scaffolded to the MBR1 genome and KUST genome using Mulit-CAR^51^. Coverage information was acquired by mapping sequencing reads back to the respective genomes. SMRT sequencing reads (15 SMRT cells) were aligned using BLASR (SMRTanalysis v.2.3.0)^37^, reporting only the best alignment for each read (“–bestn 1”). IonTorrent reads obtained in 2012 (BioProject accession: PRJEB4259, SRA accession: ERR342261) were mapped using Burrows-Wheeler Aligner (BWA 0.7^52^), employing the “mem” algorithm. The generated sequence mapping files were handled and converted as needed using SAMtools 2.1^53^ and Picard Tools 2.6.0 (http://broadinstitute.github.io/picard/). A reciprocal protein BLAST analysis between the MBR1 genome and the 2006 KUST genome was performed with BLASTP 2.4.0+^50^ using default parameters. The BLAST results were filtered on alignment length and percent identity using a custom python script followed by manual examination of results to assess strain diversity.

### Base modification analysis

All SMRT sequencing reads (15 SMRT cells, 647,491 reads) were aligned to the assembled genome. Pipelines available in SMRTanalysis v.2.3.0 were used to identify modified bases and associated motifs. DNA polymerase kinetics observed during SMRT sequencing were processed for each genomic position using a previously described protocol^27,54^. The DNA base modification analysis uses an *in silico* kinetic model and a *t*-test based scoring system to detect modified bases. The DNA did not receive Tet1 oxidation treatment prior to SMRT sequencing, therefore 5-methylcytosine signals could not be reliably detected^32^. Observations with Log-transformed P values below 100 were excluded to accurately identify 6-methyladenine and 4-methylcytosine bases. This threshold was optimized guided by the distribution of P values for different bases, minimizing the false positive rate (Supplementary Figure S6). Additional data analysis was performed using R^55^ and plotted using the Circos visualization tool^56^.

### Data availability

The corrected, whole-genome shotgun SMRT sequencing reads, assembled sequences and genome annotation for strain MBR1 are available at the European Nucleotide Archive (ENA) under study ID PRJEB22746.

## Electronic supplementary material

Supplementary materials

File S1
