# Supplementary material for: Resolving the complete genome of Kuenenia stuttgartiensis from a membrane bioreactor enrichment using Single-Molecule Real-Time sequencing
Source: Sci Rep. 2018 Mar 15;8:4580. doi: 10.1038/s41598-018-23053-7 (PMC5854607; doi:10.1038/s41598-018-23053-7)
Supplement: Supplementary file 1 — Supplementary materials [file 41598_2018_23053_MOESM1_ESM.pdf]

Supplementary information

Resolving the complete genome of *Kuenenia stuttgartiensis* from a membrane bioreactor enrichment using Single Molecule Real-Time sequencing

Jeroen Frank[1], Sebastian Lücker[2], Rolf H.A.M. Vossen[3], Mike S.M. Jetten[1,2], Richard J. Hall[4], Huub J.M. Op den Camp[2*], Seyed Yahya Anvar[3,5*]

[1]Soehngen Institute of Anaerobic Microbiology, Radboud University Nijmegen, Nijmegen, The Netherlands
[2]Department of Microbiology, IWWR, Radboud University Nijmegen, Nijmegen, The Netherlands
[3]Leiden Genome Technology Center, Leiden University Medical Center, Leiden, The Netherlands
[4]Pacific Biosciences, Menlo Park, California, United States of America
[5]Department of Human Genetics, Leiden University Medical Center, Leiden, The Netherlands

Correspondence

Microbiology
Huub J.M. Op den Camp, Department of Microbiology, IWWR, Radboud University Nijmegen.
Heyendaalseweg 135, 6525 AJ, Nijmegen, The Netherlands. h.opdencamp@science.ru.nl

Bioinformatics
Seyed Yahya Anvar, Leiden Genome Technology Center, Leiden University Medical Center.
Einthovenweg 20, 2333ZC, Leiden, The Netherlands. s.y.anvar@lumc.nl

Keywords
Kuenenia stuttgartiensis, anammox, Single Molecule Real-Time sequencing, complete genome assembly, Pacific Biosciences, membrane bioreactor, continuous enrichment culture, methylation.

Supplementary Table S1 | Read statistics of SMRT sequencing dataset.
SMRT sequencing of the enrichment culture was performed using the PacBio RS II sequencer for 14 SMRT cells. The Hierarchical Genome-Assembly Process (HGAP) pipeline (SMRTanalysis v.2.3.0) was used to correct random errors in the long, single-molecule PacBio reads. The seed length cutoff used for pre-assembly (error correction) was set to 2 Kb. After assembly, one additional SMRT cell was sequenced in an independent sequencing run to assess the accuracy and validity of the assembly and to facilitate the base modification analysis.

14 SMRT cells

	Unprocessed (filtered subreads)	Corrected (HGAP 2K)
Number of reads	540,044	108,054
Total nucleotides	1,788,961,110 bp	449,980,370 bp
Median read length	2,270 bp	2,558 bp
5th percentile	789 bp	594 bp
95th percentile	10,064 bp	12,423 bp
Maximum length	33,959 bp	27,174 bp
GC content	44.2%	41.3%

15 SMRT cells

	Unprocessed (filtered subreads)	Corrected (HGAP 6K)
Number of reads	647,491	103,316
Total nucleotides	2,839,255,068 bp	622,767,835 bp
Median read length	2,592 bp	4,756 bp
5th percentile	830 bp	568 bp
95th percentile	13,566 bp	14,633 bp
Maximum length	44,543 bp	35,264 bp
GC content	44.2%	42.0%

Supplementary Figure S1 | Assembly graph of the *de novo* assembled metagenome.
The graph below illustrates the joining of unitigs (high confidence contigs) that made up the metagenome assembly. Coverage depth is denoted by color, with high covered unitigs colored blue and low covered unitigs in red.

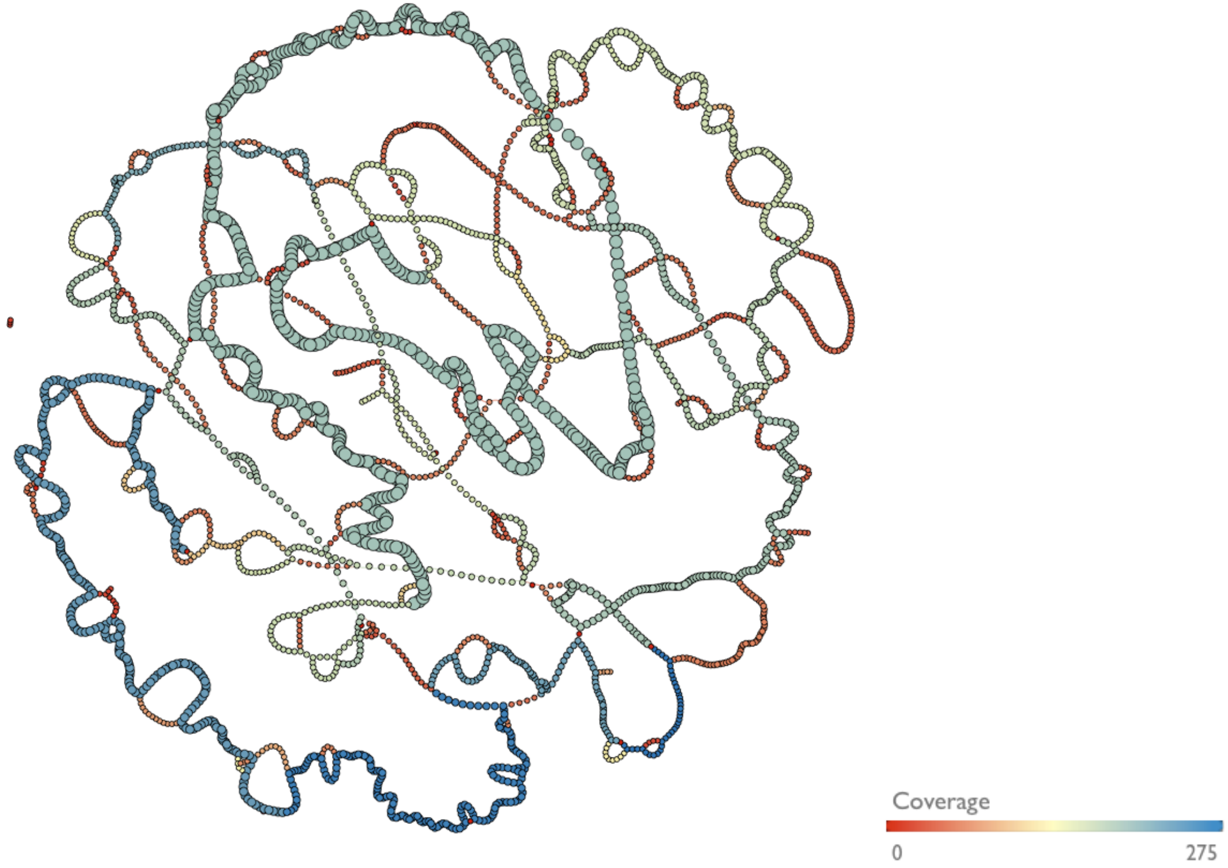

Supplementary Figure S2 | Binning reads by sequencing depth.
Uncorrected reads were mapped back to the metagenome (135 contigs) and assigned to bins based on the depth of coverage. Coverage cutoffs were set guided by the metagenome assembly graph (Supplementary Figure S1) and the depth of coverage histogram shown below. Reads aligning to extremely low covered regions (up to 25-fold) were not included in any bin, but may have contained even more low abundant *Kuenenia* diversity.

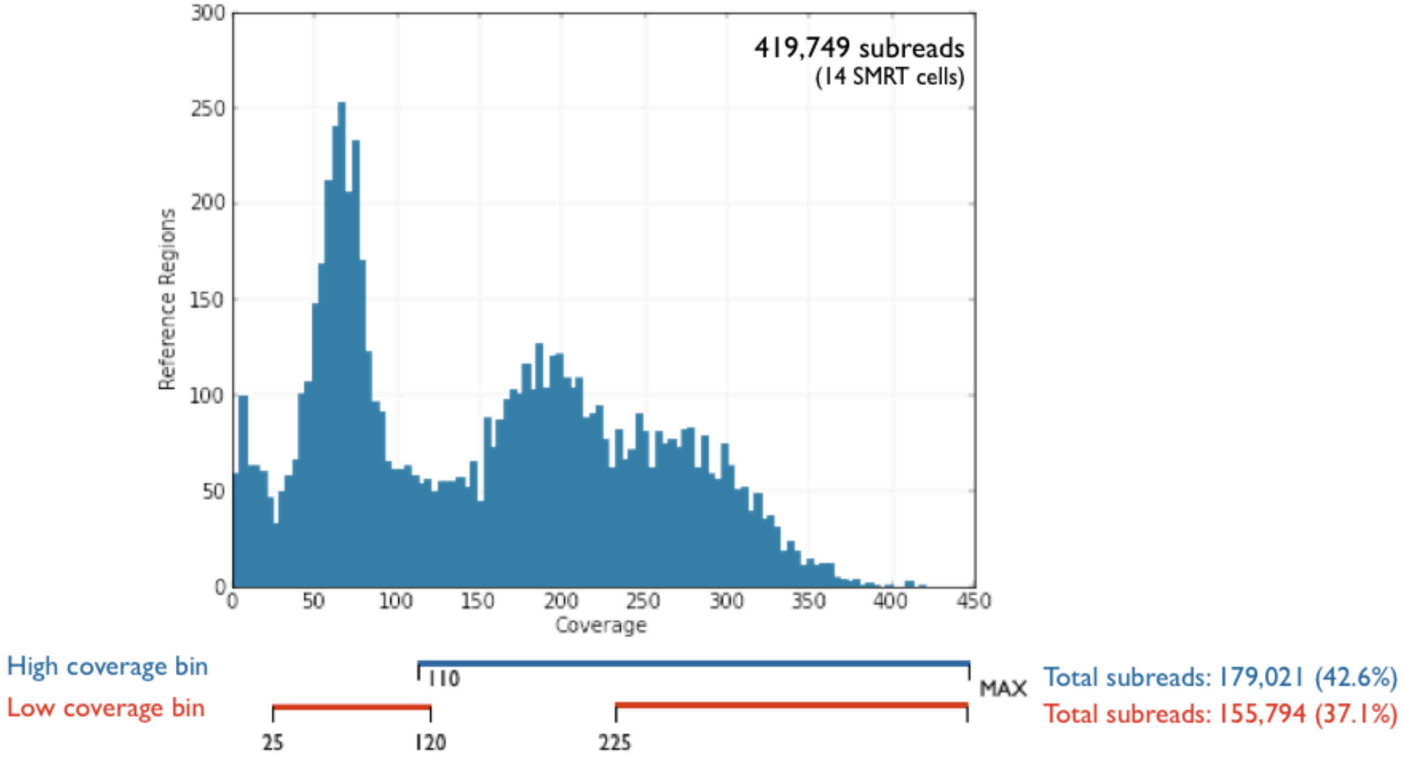

Supplementary Figure S3 | Assembly graphs of high and low coverage bins.
Reads in each bin were separately corrected (HGAP, seed cutoff 2 Kb) and assembled. The assembly of the high coverage bin was less fragmented and showed an more uniform coverage distribution compared to the metagenome assembly (Supplementary Figure S1). The low coverage assembly graph was both more complex and fragmented compared to the metagenome.

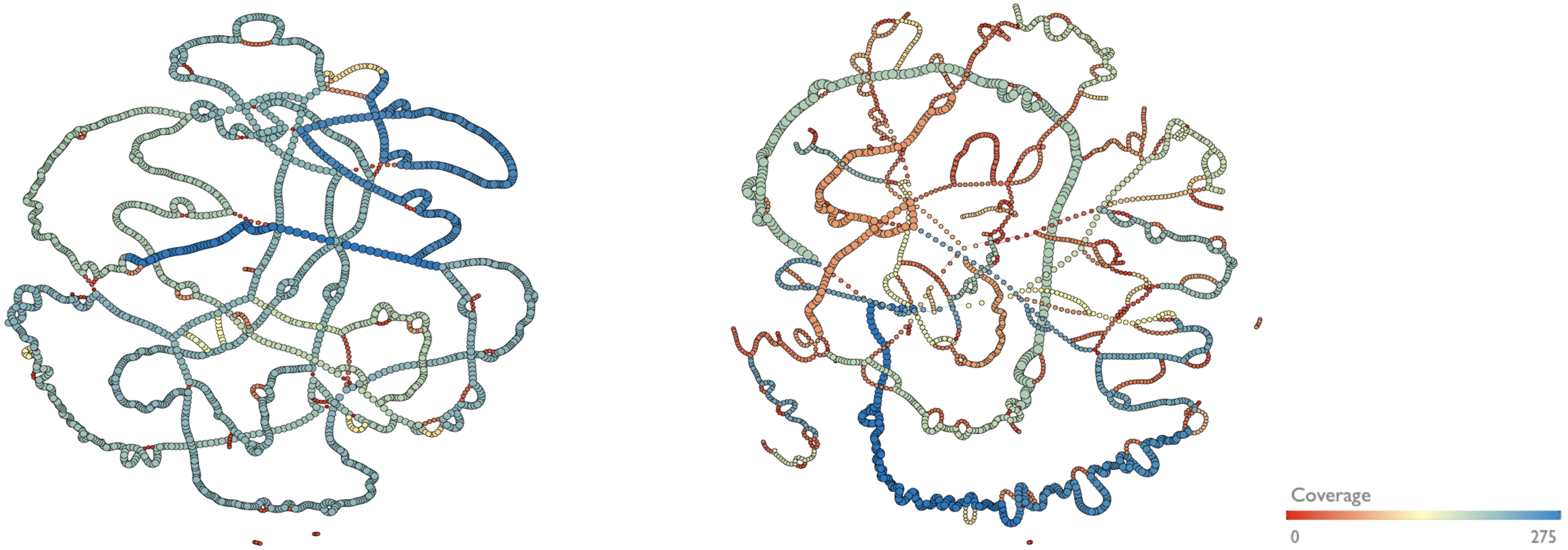

Graph high coverage bin assembly

Total contigs: 66 (5.7 Mb)

Graph low coverage bin assembly

Total contigs: 157 (7.7 Mb)

Supplementary Figure S4 | Refinement of the high coverage bin.
The high coverage bin was refined by excluding 3,085 reads that aligned to regions with <100-fold coverage in the high coverage bin assembly (Supplementary Figure S3). The remaining reads were corrected (175,936 reads (32.6%), HGAP seed length cutoff of 4 Kb) and assembled resulting in 48 contigs. The assembly graph below demonstrated the assembly was well resolved and uniform in coverage. The scatterplot indicated seven contigs (4.46 Mb) that had a markedly higher (>190-fold) coverage depth. Scaffolding and gap-filling procedures on this set of contigs ultimately yielded the complete *K. stuttgartiensis* MBR1 genome in one continuous piece.

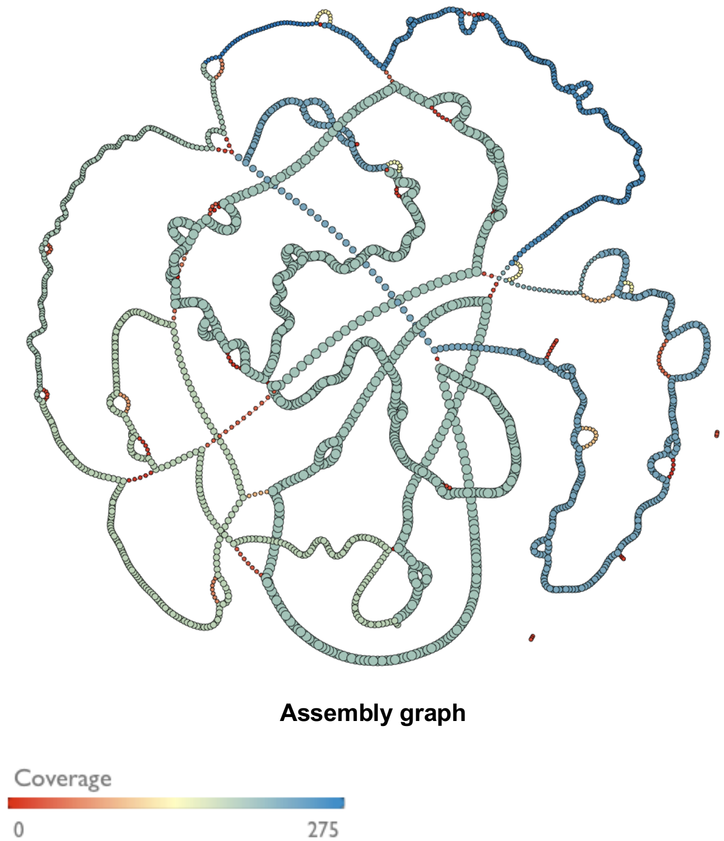

Assembly graph

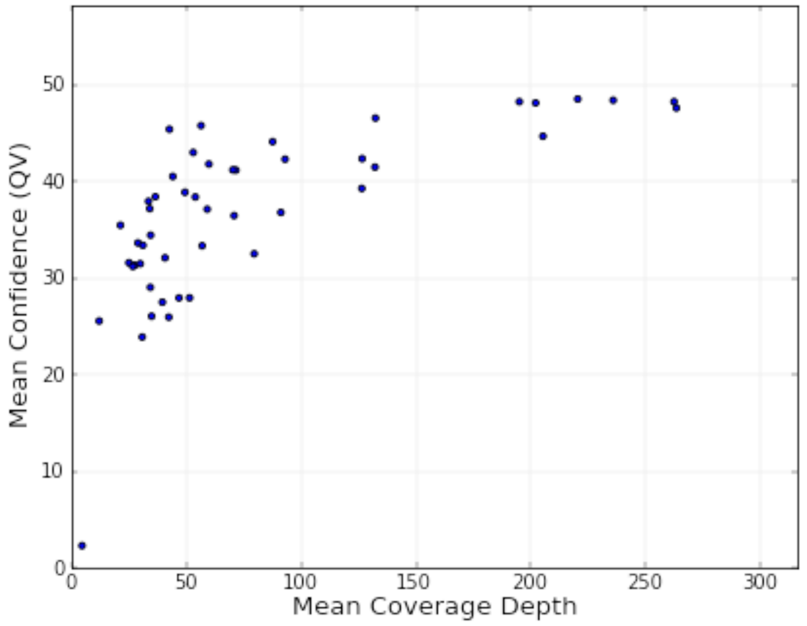

Coverage depth of assembled contigs

Supplementary Figure S5 | SMRT sequencing coverage distribution.
Uncorrected SMRT sequencing reads (14 SMRT cells, Supplementary Table 1) were aligned to the closed *K. stuttgartiensis* MBR1 genome, yielding an average coverage depth of 269x (black line). One additional SMRT cell was sequenced in an independent sequencing run to aid in the assessment of the quality of the assembly. Reads gained from this control raised the coverage on average by 150-fold (blue line). Two regions showed significantly deviating coverage levels. First, a small region at 1,118,00 – 1,120,00 had markedly lower total coverage depth varying between ~110x and ~220x (15 SMRT cells). Two transposases are located in this region. Second, at location 1,692,00 – 1,722,00, total coverage shifted upwards fluctuating from ~650x to ~900x (15 SMRT cells). A genomic island is predicted at this location (Island GI11, 1,699,82 – 1,711,813, Supplementary Table S2).

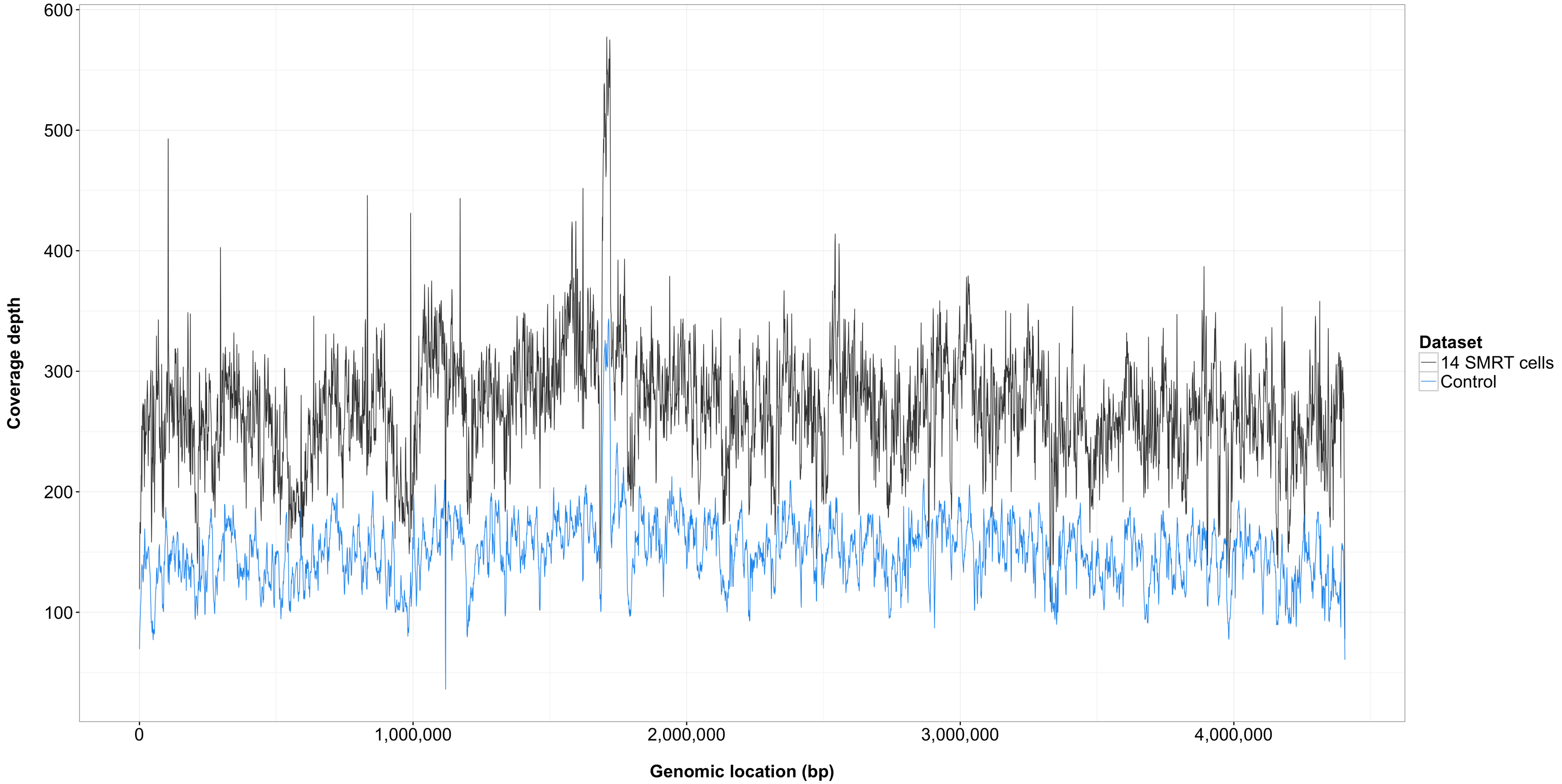

Supplementary Table S2 | Genomic islands in *Kuenenia stuttgartiensis* MBR1.
Overview of putative genomic islands (GIs) predicted by IslandViewer 3 (Dhillon et al. *Nucleic Acids Research* 43 (2015)). The GC% column denotes the percentage GC content of the GI including its deviation from the genome wide mean (41.1%).

ID	Start	End	Size (bp)	GC%	Genes
GI1	43,320	57,384	14,064	45.8 (+4.7)	KSMBR1_0036 - KSMBR1_0057 (22)
GI2	84,525	90,502	5,977	44.1 (+3.0)	KSMBR1_0084 - KSMBR1_0093 (10)
GI3	201,084	208,428	7,344	45.7 (+4.6)	KSMBR1_0207 - KSMBR1_0218 (12)
GI4	508,871	515,430	6,559	43.9 (+2.8)	KSMBR1_0483 - KSMBR1_0492 (10)
GI5	544,452	564,728	20,276	45.4 (+4.3)	KSMBR1_0516 - KSMBR1_0525 (10)
GI6	576,936	596,744	19,808	42.0 (+0.9)	KSMBR1_0537 - KSMBR1_0546 (10)
GI7	990,343	997,867	7,524	37.9 (- 3.2)	KSMBR1_0917 - KSMBR1_0927 (11)
GI8	1,200,431	1,206,074	5,643	45.8 (+4.7)	KSMBR1_1112 - KSMBR1_1121 (10)
GI9	1,211,261	1,231,921	20,660	45.3 (+4.2)	KSMBR1_1127 - KSMBR1_1137 (11)
GI10	1,322,779	1,332,700	9,921	46.9 (+5.8)	KSMBR1_1222 - KSMBR1_1238 (17)
GI11	1,699,820	1,711,813	11,993	49.2 (+8.1)	KSMBR1_1575 - KSMBR1_1583 (9)
GI12	2,154,381	2,160,217	5,836	37.7 (- 3.4)	KSMBR1_1973 - KSMBR1_1983 (11)
GI13	2,221,289	2,229,092	7,803	36.3 (- 4.8)	KSMBR1_2035 - KSMBR1_2046 (12)
GI14	2,322,149	2,334,352	12,203	43.4 (+2.3)	KSMBR1_2128 - KSMBR1_2143 (16)
GI15	2,469,220	2,472,810	3,590	40.9 (- 0.2)	KSMBR1_2265 - KSMBR1_2273 (9)
GI16	2,473,897	2,482,267	8,370	38.1 (- 3.0)	KSMBR1_2276 - KSMBR1_2290 (15)
GI17	2,519,513	2,523,054	3,541	39.9 (- 1.2)	KSMBR1_2335 - KSMBR1_2344 (10)
GI18	2,881,739	2,887,378	5,639	45.6 (+4.5)	KSMBR1_2673 - KSMBR1_2682 (10)
GI19	3,336,176	3,342,689	6,513	44.1 (+3.0)	KSMBR1_3108 - KSMBR1_3118 (11)
GI20	3,488,411	3,500,150	11,739	43.4 (+2.3)	KSMBR1_3250 - KSMBR1_3262 (13)
GI21	3,674,637	3,679,977	5,340	38.2 (- 2.9)	KSMBR1_3406 - KSMBR1_3415 (10)
GI22	3,942,666	3,967,367	24,701	48.0 (+6.9)	KSMBR1_3653 - KSMBR1_3670 (15)
GI23	3,970,879	3,978,056	7,177	45.1 (+4.0)	KSMBR1_3674 - KSMBR1_3684 (11)
GI24	4,156,621	4,164,291	7,670	44.5 (+3.4)	KSMBR1_3848 - KSMBR1_3858 (11)
GI25	4,185,716	4,217,054	31,338	42.7 (+1.6)	KSMBR1_3878 - KSMBR1_3907 (30)
GI26	4,328,174	4,334,840	6,666	42.2 (+1.1)	KSMBR1_4008 - KSMBR1_4016 (9)
GI27	4,389,752	4,398,947	9,195	44.1 (+3.0)	KSMBR1_4075 - KSMBR1_4087 (13)

Supplementary Table S3 | CRISPRs found in *Kuenenia stuttgartiensis* MBR1.
CRISPRs identified by the CRISPRFinder web tool (Grissa et al. *Nucleic Acids Research*. 35 (2007)). The location reported by CRISPRFinder sometimes deviated slightly from the annotation generated by Prokka 1.10 (marked with asterisk) (Seemann *Bioinformatics* 30 (2014).

Start	End	Size (bp)	Direct Repeat (DR) consensus sequence	No. spacers
*93,784	*94,427	643	GTCTCAATCCCTTCTACATCAGGTCAATGATTTCCAC	8
109,130	109,914	784	GTCTCAATCCCTTCTACATCAGGTCAATGATTTCCAC	10
*489,976	490,524	548	GTTTCAATTCCTTATAGGTGCAATGAGAC	8
*492,068	493,063	995	GTTTCAATTCCTTATAGGTGCAATGAGAC	15
495,108	496,174	1,066	GTTTCAATTCCTTATAGGTGCAATGAGAC	16
498,283	498,766	483	GTTTCAATTCCTTATAGGTGCAATGAGAC	7
296,2833	296,9923	7,090	GTTTCAATTCCTCATAGGTAGAATGAAAAC	106

Supplementary Figure S6 | Comparison of *Kuenenia* assembles.
The OrthoANI method was used to calculate the average nucleotide identity (ANI) for every assembly. Genomes were aligned using NUCmer (MUMmer 3 – Kurtz et al. *Genome boil.* 5 (2004)). Alignments ≥ 500 bp with an average nucleotide identity of ≥ 95% were considered.

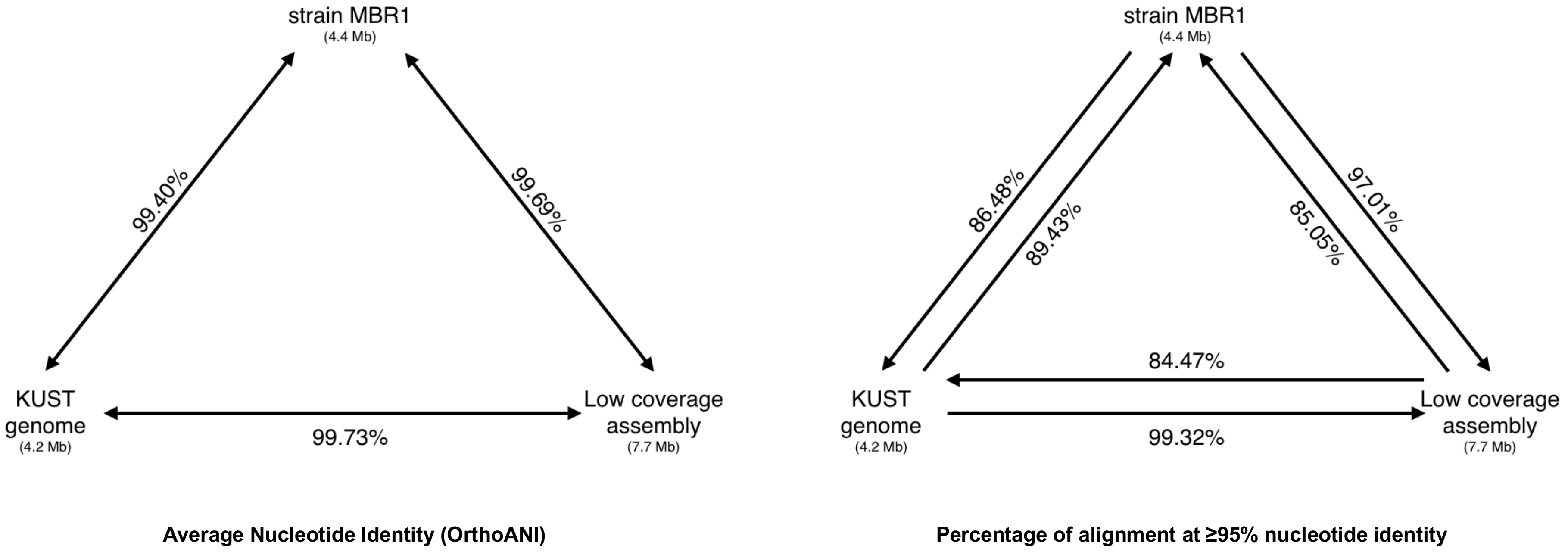

Supplementary Figure S7 | Reciprocal BLASTP results for the MBR1 strain and the KUST genome.
Bar graph illustrating the number of proteins without a significant BLASTP hit in one genome versus the other (light green: MBR1 strain, dark green: KUST genome). A set of rules was used to exclude small and low identity alignments. First, query coverage must fall within 25%-120% (left), 50-120% (middle) or 75-120% (right). Allowing alignments up to 120% of the query length accounts for gaps, small changes in gene length and other differences. Second, a cutoff for the amino acid percent identity was applied (indicated on the x-axis).

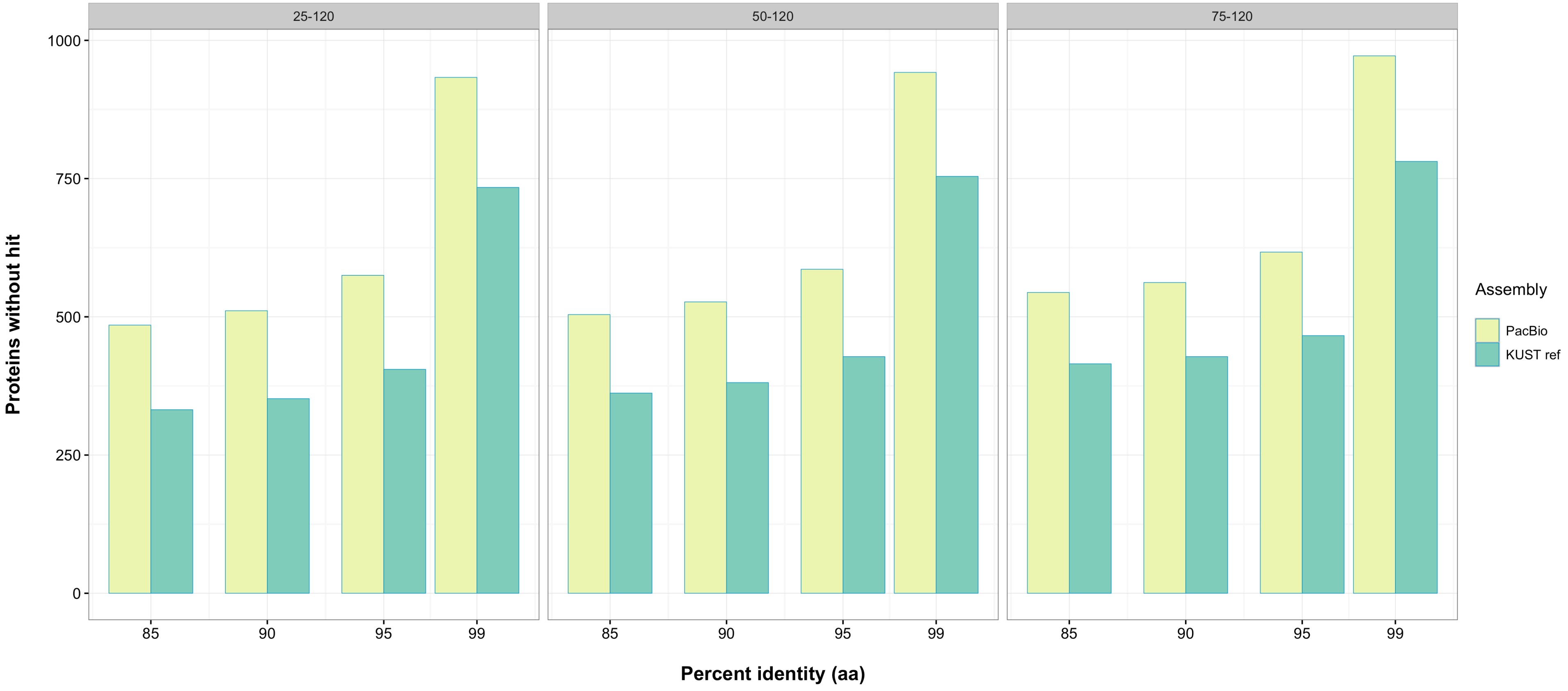

Supplementary Table S4 | Components of restriction-modification systems in *Kuenenia stuttgartiensis* MBR1.

Locus tag	Start	End	Strand	Annotation	Gene	EC number	KUST (%AAI)
KSMBR1_1845	2,005,662	2,007,116	+	similar to type I restriction modification enzyme M chain	hsdM_1	2.1.1.72	99
KSMBR1_1847	2,008,143	2,009,303	+	similar to type I restriction modification enzyme S chain	hsdS_1	3.1.21.3	99
KSMBR1_1875	2,043,933	2,047,364	-	similar to type IIS restriction/modification enzyme; site-specific DNA methyltransferase (adenine specific)	NA	2.1.1.72	-
KSMBR1_1952	2,134,686	2,135,291	+	similar to type IIS restriction/modification enzyme; site-specific DNA methyltransferase (adenine specific)	NA	2.1.1.72	-
KSMBR1_2048	2,231,113	2,234,919	-	similar to type IIS restriction/modification enzyme; site-specific DNA methyltransferase (adenine specific)	NA	2.1.1.72	-
KSMBR1_2450	2,626,894	2,630,628	+	similar to type IIS restriction/modification enzyme; site-specific DNA methyltransferase (adenine specific)	NA	2.1.1.72	-
KSMBR1_2551	2,745,325	2,746,539	+	Type I restriction modification DNA specificity domain protein	NA	NA	-
KSMBR1_2553	2,747,064	2,748,410	+	similar to type I restriction modification enzyme M chain	hsdM_2	2.1.1.72	-
KSMBR1_3272	3,514,502	3,516,241	+	similar to type IIS restriction/modification enzyme; site-specific DNA methyltransferase (adenine specific)	NA	2.1.1.72	-

Supplementary Table S5 | DNA and RNA methyltransferases in *Kuenenia stuttgartiensis* MBR1.

Locus tag	Start	End	Strand	Annotation	Gene	EC number	KUST (%AAI)
KSMBR1_0006	6,636	7,622	+	similar to DNA-methyltransferase (cytosine-specific)	NA	2.1.1.113	-
KSMBR1_0292	275,202	276,041	+	DNA adenine methylase	dam_1	2.1.1.72	-
KSMBR1_0532	572,244	573,002	-	Methyltransferase domain protein	NA	NA	-
KSMBR1_0547	597,152	598,069	-	Methyltransferase domain protein	NA	NA	-
KSMBR1_0811	876,028	876,708	-	strongly similar to tRNA (guanine-N(1)-)-methyltransferase	trmD	NA	100
KSMBR1_0835	899,669	900,562	+	Modification methylase DpnIIA	dpnM	2.1.1.72	-
KSMBR1_0896	965,706	966,218	-	tRNA (mo5U34)-methyltransferase	cmoB	2.1.1.-	-
KSMBR1_1445	1,562,130	1,562,864	-	similar to RNA methyltransferase YggJ	yggJ	2.1.1.-	100
KSMBR1_1875	2,043,933	2,047,364	-	similar to type IIS restriction/modification enzyme; site-specific DNA methyltransferase (adenine specific)	NA	2.1.1.72	-
KSMBR1_1952	2,134,686	2,135,291	+	similar to type IIS restriction/modification enzyme; site-specific DNA methyltransferase (adenine specific)	NA	2.1.1.72	-
KSMBR1_2048	2,231,113	2,234,919	-	similar to type IIS restriction/modification enzyme; site-specific DNA methyltransferase (adenine specific)	NA	2.1.1.72	99
KSMBR1_2207	2,413,873	2,414,193	+	similar to HhaI Dna (cytosine-C5-)-methyltransferase	dcm	2.1.1.37	94
KSMBR1_2364	2,536,922	2,537,485	-	strongly similar to methylated-DNA-protein-cysteine S-methyltransferase	ogt	2.1.1.63	99
KSMBR1_2450	2,626,894	2,630,628	+	similar to type IIS restriction/modification enzyme; site-specific DNA methyltransferase (adenine specific)	NA	2.1.1.72	-
KSMBR1_2616	2,816,930	2,818,510	+	similar to DNA-methyltransferase (cytosine-specific)	NA	2.1.1.113	97
KSMBR1_3195	3,418,443	3,419,549	+	similar to adenine-specific DNA methylase	dam_2	2.1.1.72	100
KSMBR1_3272	3,514,502	3,516,241	+	similar to type IIS restriction/modification enzyme; site-specific DNA methyltransferase (adenine specific)	NA	2.1.1.72	99
KSMBR1_3727	4,021,545	4,023,326	-	similar to adenine specific DNA methylase	mod	2.1.1.72	-
KSMBR1_4048	4,367,732	4,368,340	-	N-6 DNA Methylase	NA	NA	100

Supplementary Figure S8 | Base modification quality values (modQVs) of modified bases in the *Kuenenia stuttgartiensis* MBR1 genome. The base modification analysis was performed using the complete SMRT sequencing dataset (15 SMRT cells). Quality values above an expected threshold are compared to the strand-specific coverage at each genomic position. The average quality values for the modification of each base were used to estimate the overall distribution and to determine an appropriate threshold for reliable identification of methylated bases (modQV or “score” ≥100).

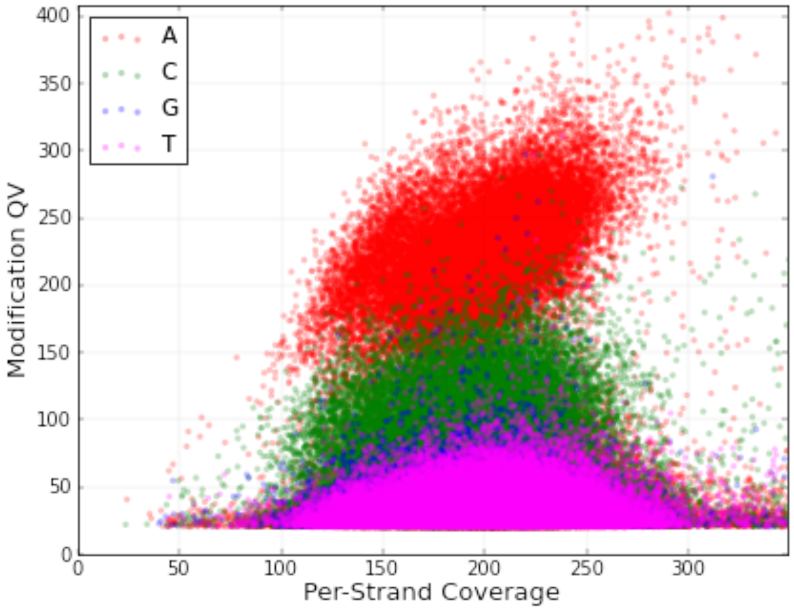

Modification quality values compared to coverage per strand

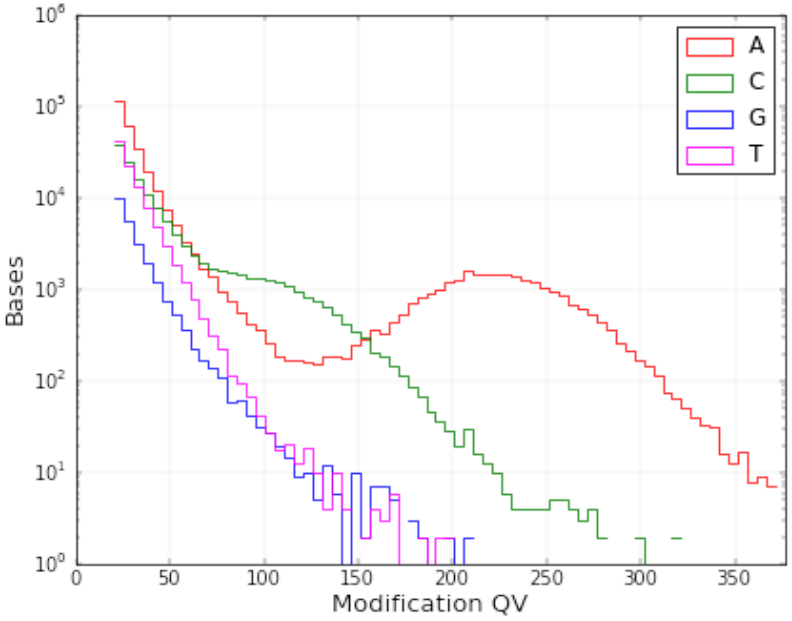

Modification quality value distributions per base

Supplementary Figure S9 | IonTorrent and SMRT sequencing reads mapping to strain specific genes of MBR1 and the KUST genome. Every data point corresponds to a gene exclusive to MBR1 (562, blue) or the KUST genome (428, red). The axes indicate the number of reads with a BLASTN hit to a gene (x-axis: SMRT sequencing, y-axis: IonTorrent). Small and low identity hits (≤97%) were excluded.

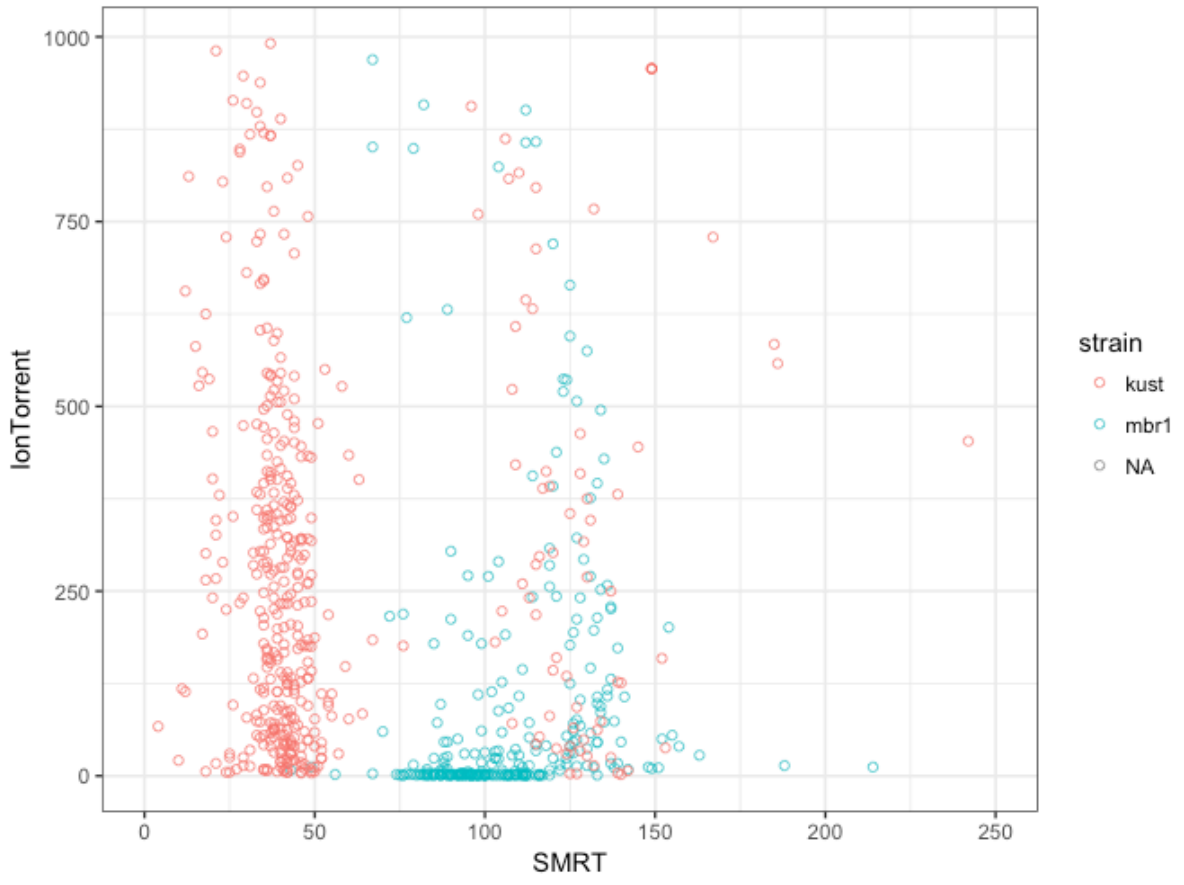

Supplementary Data S1 | Celera assembler 8.1 configuration settings.
Configuration settings ("spec file") for *de novo* assembly of corrected SMRT sequencing reads using Celera assembler 8.1. Settings controlling the performance and optimization of computing resources have been omitted.

```
merSize                    = 14
overlapper                 = ovl
ovlMinLen                  = 40
unitigger                  = bogart
utgBubblePopping           = 1
doToggle                   = 0
toggleNumInstances         = 0
toggleUnitigLength         = 2000
doOverlapBasedTrimming     = 1
doExtendClearRanges        = 2
cgwDemoteRBP               = 0
cgwMergeMissingThreshold   = 0
```
